# Identification and analysis of differentially expressed lncRNAs and their ceRNA networks in DMD/mdx primary myoblasts

**DOI:** 10.1038/s41598-024-75221-7

**Published:** 2024-10-10

**Authors:** Abdolvahab Ebrahimpour Gorji, Kasra Ahmadian, Zahra Roudbari, Tomasz Sadkowski

**Affiliations:** 1 Department of Physiological Sciences, Institute of Veterinary Medicine, Warsaw University of Life Sciences, Warsaw, 02-776 Poland; 2 Department Animal Science, Faculty of Agriculture, Ferdowsi University of Mashhad, Mashhad, Iran; 3 Department of Animal Science, Faculty of Agriculture, University of Jiroft, Jiroft, Iran

**Keywords:** Gene expression analysis, Muscle stem cells, Epigenomics

## Abstract

This study explored the significance of long non-coding RNAs (lncRNAs), particularly their role in maintaining dystrophin protein stability and regulating myocyte proliferation and differentiation. The investigation focused on DMD/mdx mouse skeletal muscle primary myoblasts, aiming to identify lncRNAs potential as biomarkers and therapeutic targets for Duchenne muscular dystrophy (DMD). Utilizing CLC Genomics Workbench software, 554 differentially expressed lncRNAs were identified in DMD/mdx mice compared to wild-type (WT) control. Among them, 373 were upregulated, and 181 were downregulated. The study highlighted specific lncRNAs (e.g., 5930430L01Rik, Gm10143, LncRNA1490, LncRNA580) and their potential regulatory roles in DMD key genes like *IGF1*, *FN1*, *TNNI1*, and *MYOD1*. By predicting miRNA and their connections with lncRNA and mRNA (ceRNA network) using tools such as miRNet, miRSYSTEM and miRCARTA, the study revealed potential indirect regulation of *Dystrophin*, *IGF1R* and *UTRN* genes by identified lncRNAs (e.g. 2310001H17Rik-203, C130073E24Rik-202, LncRNA2767, 5930430L01Rik and LncRNA580). These findings suggest that the identified lncRNAs may play crucial roles in the development and progression of DMD through their regulatory influence on key gene expression, providing valuable insights for potential therapeutic interventions.

**Supplementary Information:**

The online version contains supplementary material available at 10.1038/s41598-024-75221-7.

## Introduction

Long non-coding RNAs (lncRNAs) are a group of transcripts bigger than 200 nucleotides in length without protein-coding capacity. They are transcribed by the non-coding regions of the animal and human genome and various parts of genomic regions, such as exonic, intronic, and intergenic^1,2^. LncRNAs are divided according to their relative position with the genetic loci, such as long intergenic non-coding RNAs (lincRNAs), intronic lncRNAs, antisense lncRNAs, and enhancer RNAs (eRNAs)^1^. LncRNAs regulate gene expression in different ways at epigenetic, transcriptional, chromatin remodeling, and translation levels^2^. The expression profiles of lncRNAs depend on the type of cell, the stage of development, and the cell’s physiological conditions^1^.

LncRNAs play a significant role in various diseases, especially cardiovascular diseases, serving as valuable biomarkers for the diagnosis and prognosis of illnesses such as myocardial infarction, heart failure, and atherosclerosis^3–5^. These lncRNAs actively regulate gene expression and influence cell signaling pathways in the vascular wall, thereby influencing endothelial dysfunction, vascular smooth muscle cell phenotypes, and leukocyte activation^6^. A recent study evaluating heart tissue from individuals with heart failure (HF) and healthy donors revealed that the lncRNA COL1A1, previously linked to fibrosis, was significantly associated with HF progression^7^. Furthermore, the study showed that elevated plasma COL1A1 levels were correlated with decreased survival rate within one year after heart transplantation in patients with HF. Additionally, the presence of lncRNA H19 in plasma was found to be an indicator of the severity and prognosis of pulmonary arterial hypertension^8^. These findings suggest that plasma lncRNA levels may serve as markers to recognize the malignant progression of HF. Moreover, another study examined the correlation between elevated lncRNA H19 levels in peripheral blood mononuclear cells and the risk of coronary artery disease (CAD) and found that lncRNA H19 levels were elevated in CAD patients compared with healthy controls. The authors suggest that this lncRNA may potentially become a biomarker of the atherosclerotic process^9^.

LncRNA also plays a crucial role in skeletal muscle differentiation and regeneration. According to the Chen et al.^10^ study, lncRNA Has2os is highly expressed in skeletal muscle, significantly upregulated during differentiation, and regulates this process by inhibiting the JNK/MAPK signaling pathway. Another study shows that changes in the expression of lncRNAs between the developmental stages of myoblast proliferation and differentiation are closely related to chromatin states^11^. In addition, another study identified lncIRS1 as a novel lncRNA that regulates myoblast proliferation and differentiation in vitro, muscle mass, and mean muscle fiber in vivo^12^. Finally, a study by Alession et al.^13^ demonstrated that the lncRNA Pvt1 is activated early during muscle atrophy, impacts mitochondrial respiration and morphology, and affects mito/autophagy, apoptosis, and myofiber size in vivo. These findings suggest that lncRNAs have an important role in skeletal muscle development and atrophy, providing a basis for the lncRNA-related therapy of muscle diseases.

An X-linked myopathy known as Duchenne muscular dystrophy (DMD) is characterized by progressive skeletal muscle weakness combined with dilated cardiomyopathy. Mutations in the *Dystrophin* gene result in protein dysfunction^14^. The lncRNAs responsible for dystrophin protein stabilization may represent promising therapeutic targets and/or biomarkers of DMD. The study of Gargaun et al.^15^ analyzed the presence of genomic lncRNA in 38 patients with Becker muscular dystrophy (BMD). It characterized the lncRNA in introns 44 and 55 of the *Dystrophin* gene. The study indicated some lncRNAs differentially expressed during myogenesis in immortalized and primary human myoblasts. One of them, the lncRNA44s2, was pointed out as a possible accelerator of differentiation. Interestingly, lncRNA44s2 expression was associated with a dystrophic phenotype. These findings suggest that it may be involved in the regulation of the muscle differentiation process and become a potential biomarker of disease progression. Based on these results, the study supports Multi-Exon Skipping (MES) therapy and proposes that the CRISPR/Cas9 MES45-55 assay design consider the lncRNA sequences bordering the exonic 45 to 55 deletion^15^.

For a better understanding of the role of lncRNA in DMD disease, the present analysis was performed aiming to investigate the role of lncRNA expression levels in the DMD/mdx mouse model and provide some clues as to the mechanism of lncRNA regulation in myogenesis and skeletal muscle atrophy in DMD.

## Results

### Assembly, annotation, and lncRNA mining

 A brief description of the lncRNA reads, assembly, and mining data is shown in Table 1. The high-throughput sequencing technique provided an average of 65,143,212 raw reads for each sample, and 99.9% of the raw reads passed the quality control. After the *de novo* assembly, 27,674 contigs were obtained with an average length of 10,240 bp. Of these contigs, 56.56% (15,653) were identified as possible lncRNAs with an average length of 16,504 bp. Among these potential lncRNAs, 3177 (20.30%) were mapped on the *Mus musculus* genome as known lncRNA, while 12,476 (79.70%) were not recognized, unknown lncRNA.

**Table 1:** Summary of the Illumina sequencing, assembly, and lncRNA identification.

Reads	
Total reads	390,859,276
Mean reads per sample	65,143,212
Mean trimmed reads	99.99%
De Novo Assembly	
Contigs	27,674
Minimum length	250 bp
Maximum length	20,231 bp
Average length	10,240 bp
LncRNAs	
Potential lncRNAs	15,653 (56.56%)
Average length	16,504 bp
Known lncRNAs	3177 (20.30%)
Unknown lncRNAs	12,476 (79.70%)

### Differential expression of lncRNAs

**Fig. 1:**
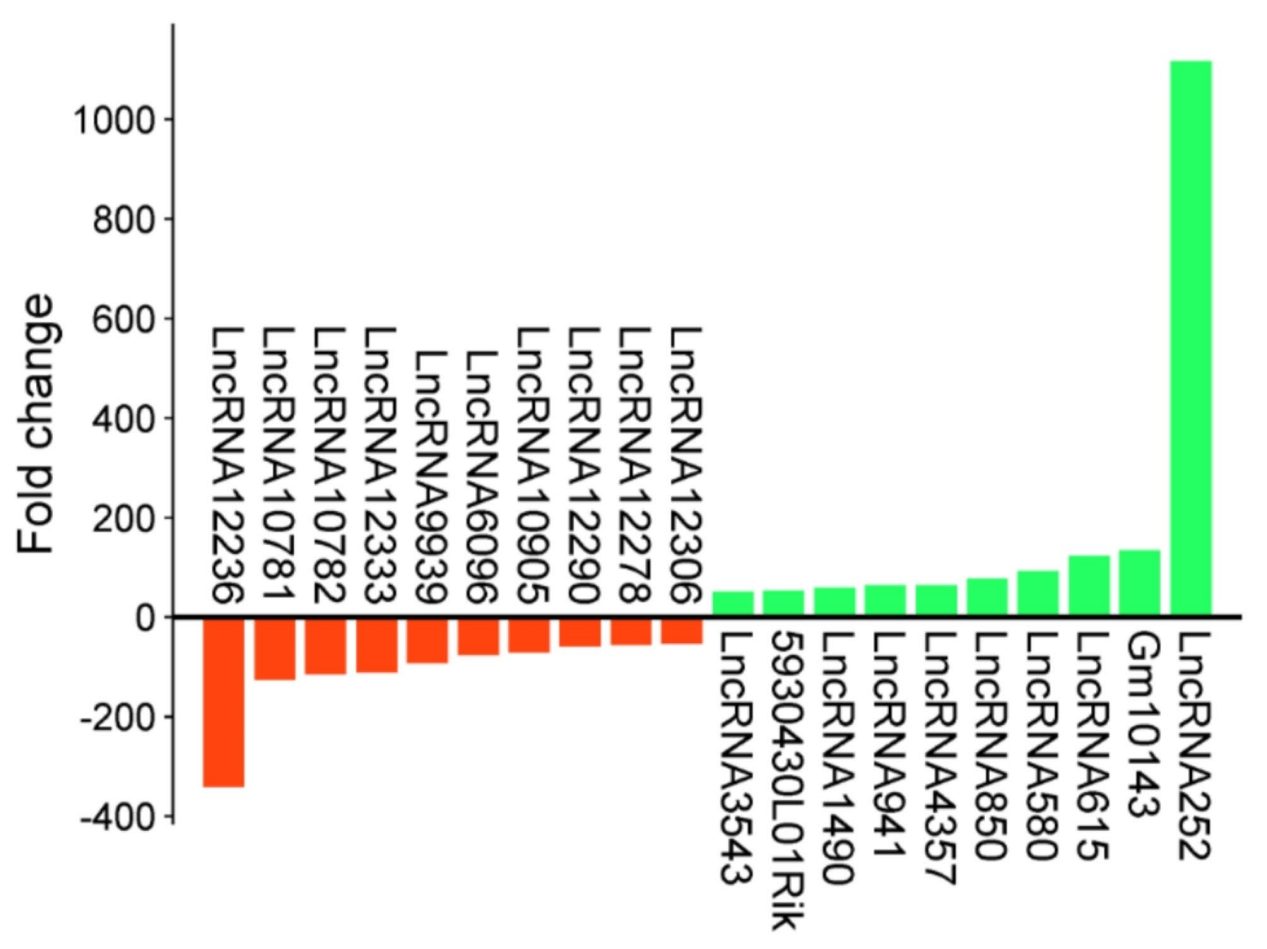
Top 20 identified lncRNAs (downregulated—red; upregulated—green).

 The differential expression analysis was conducted on the DMD/mdx primary myoblast and wild type (WT) control samples with the following criteria: fold change (FC) greater than the absolute value of 2 and a p-value < 0.05. Ultimately, we generated a file comprising 15,653 sequences identified as potential lncRNA candidates. The results of this analysis are provided in Supplementary file S1. In the DMD/mdx myoblasts, the analysis led to the highest modulation of lncRNAs, resulting in 554 differentially expressed (DE) lncRNAs (Supplementary file S1). Among them, 373 lncRNAs were upregulated, while 181 lncRNAs were downregulated. The top 10 upregulated and downregulated lncRNAs are shown in Fig. 1. These lncRNAs were ranked based on their fold change values and significance in the DMD/mdx myoblasts compared to the WT.

### MiRNA prediction

 Using the psRNATarget web server, we identified potential interactions between lncRNAs and miRNAs, shedding light on the intricate regulatory relationships between these molecules. This analysis provides valuable insights into the mechanisms through which lncRNAs can influence gene expression via miRNA-mediated regulation. Our investigation successfully predicted 1,750 miRNAs for upregulated lncRNAs and 1,297 miRNAs for downregulated lncRNAs. To delve deeper into the analysis, we focused on the top 10 upregulated and downregulated lncRNAs, investigating their specific interactions with miRNAs for further study. We found 287 miRNA for upregulated and 78 miRNA for downregulated top lncRNAs (Supplementary file S2).

### Network analysis—Hub genes

 Based on the gene expression analysis conducted by Gosselin et al.^16^, 170 genes were identified as differentially expressed between DMD/mdx and WT primary mouse myoblasts^16^. Among these, 105 genes were downregulated, while 65 genes were upregulated, using log2 fold change (log2FC) and an adjusted p-value cutoff of > 0.05. To better understand the regulatory network associated with the differentially expressed genes, we utilized the powerful CytoHubba plugin in Cytoscape. By employing this software tool, we compared the results (20 top genes) obtained from various algorithms (betweenness, closeness, DMNC, MCC, degree), enabling us to identify 14 common hub genes across applied different algorithms. Among hub genes, several notable ones emerged as critical players in the regulatory network. These include actin, alpha cardiac muscle 1 (*ACTA1)*, insulin-like growth factor 1 *(IGF1)*, fibronectin 1 *(FN1)*, troponin I *(TNNI1)*, myogenin *(MYOG)*, ATPase sarcoplasm-ic/endoplasmic reticulum Ca2 + transporting 1 *(ATP2A1)*, creatine kinase, muscle type *(CKM)*, troponin T1 *(TNNT1)*, actinin alpha 3 *(ACTN3)*, myogenic differentiation 1 *(MYOD1)*, troponin T3 *(TNNT3)*, myosin light chain *(MYLPF)*, troponin T2 *(TNNT2)*, and myoglobin *(MB)* (Table 2).

**Table 2:** The top 20 nodes are ranked by the betweenness, closeness, DMNC, MCC, and degree methods.

Betweenness			Closeness			DMNC			MCC			Degree		
Rank	Gene	Score	Rank	Gene	Score	Rank	Gene	Score	Rank	Gene	Score	Rank	Gene	Score
1	*FN1*	2690.71	1	*ACTA1*	61.51	1	*TNNI1*	0.87045	1	*TNNT3*	2.63E+08	1	*ACTA1*	26
2	*H4C11*	2050.02	2	*FN1*	59.56	2	*TNNC1*	0.86152	2	*TNNI2*	2.63E+08	2	*TNNT3*	24
3	*MB*	1556.18	3	*TNNT2*	58.86	3	*MYL4*	0.85126	3	*ACTA1*	2.62E+08	3	*CASQ2*	22
4	*MYOG*	1264.68	4	*TNNT3*	58.16	4	*DES*	0.84357	4	*ACTN3*	2.62E+08	3	*ACTN3*	22
5	*ACTA1*	1225.47	5	*MB*	57.66	5	*ACTN3*	0.79728	5	*TNNT2*	2.61E+08	3	*TNNT2*	22
6	*IGF1*	1221.61	6	*MYOG*	57.45	6	*MYH3*	0.79697	6	*TNNI1*	2.45E+08	3	*FN1*	22
7	*CKM*	1109.47	7	*MYOD1*	57.36	7	*TNNI2*	0.79413	7	*TNNT1*	2.44E+08	3	*ACTC1*	22
8	*ENO3*	1008.08	8	*CKM*	57	8	*TNNT1*	0.78860	8	*MYLPF*	2.21E+08	8	*MYLPF*	21
9	*MYOD1*	830.23	9	*MYH3*	56.55	9	*ATP2A1*	0.78338	9	*MYOG*	1.75E+08	8	*MYOG*	21
10	*CAV3*	668.36	10	*ACTN3*	56.41	10	*TNNT3*	0.77985	10	*MYOD1*	1.74E+08	10	*MB*	20
11	*TNNT3*	609.96	11	*TNNI2*	56	11	*ENO3*	0.77178	11	*ACTC1*	1.73E+08	11	*CKM*	19
12	*TNNT2*	584.57	12	*CAV3*	55.53	12	*MB*	0.76771	12	*MYH3*	1.69E+08	11	*MYOD1*	19
13	*SYNPO2L*	456.05	13	*ACTC1*	55.36	13	*PDLIM3*	0.76638	13	*MB*	9.29E+07	11	*TNNT1*	19
14	*MYH3*	436.02	14	*MYLPF*	55	14	*MYLPF*	0.76575	14	*ATP2A1*	8.97E+07	14	*SRL*	18
15	*CCK*	410	15	*SRL*	54.26	15	*TNNT2*	0.76064	15	*TNNC1*	8.53E+07	14	*TNNI1*	18
16	*MUSK*	408	16	*ATP2A1*	54.16	16	*H4C14*	0.73986	16	*MYL4*	8.03E+07	16	*ATP2A1*	17
17	*ADH1*	408	17	*CASQ2*	54.13	17	*KLHL41*	0.73197	17	*DES*	4.05E+07	17	*IGF1*	16
18	*–*	326.41	18	*TNNT1*	53.83	18	*H4C6*	0.71829	18	*CKM*	1.73E+07	18	*TNNC1*	14
19	*COL2A1*	319.62	19	*TNNI1*	53.03	19	*H4C17*	0.71599	19	*SRL*	2,589,924	19	*SYNPO2L*	13
20	*ITGA7*	304	20	*IGF1*	51.45	19	*H2AC11*	0.71599	20	*ENO3*	977,792	20	*Pdlim3*	12

**Fig. 2:**
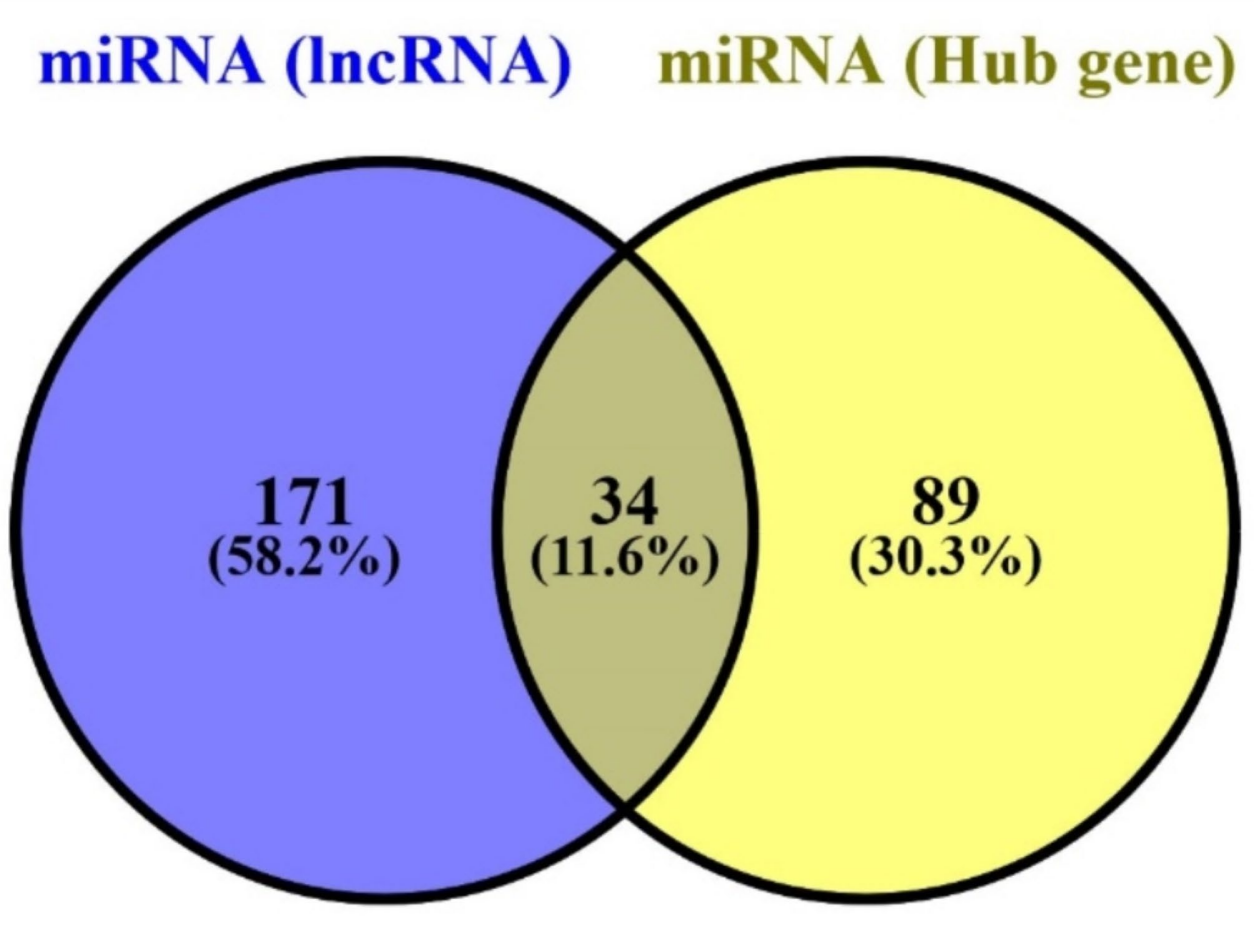
MiRNAs shared between the lncRNA and hub genes.

Following the identification of hub genes, we utilized three different software tools, miRWalk, miRCARTA, and miRSYSTEM, to predict potential miRNA targets for them. These tools leverage sequence complementarity and other criteria to predict putative binding sites between miRNAs and target genes. Finally, we compared the miRNAs predicted to target the hub genes with those predicted to target the lncRNAs of interest. This comparison aimed to identify common miRNAs between the two datasets, providing a basis for further analysis (Fig. 2).

### LncRNA-miRNA-mRNA regulatory network

**Fig. 3:**
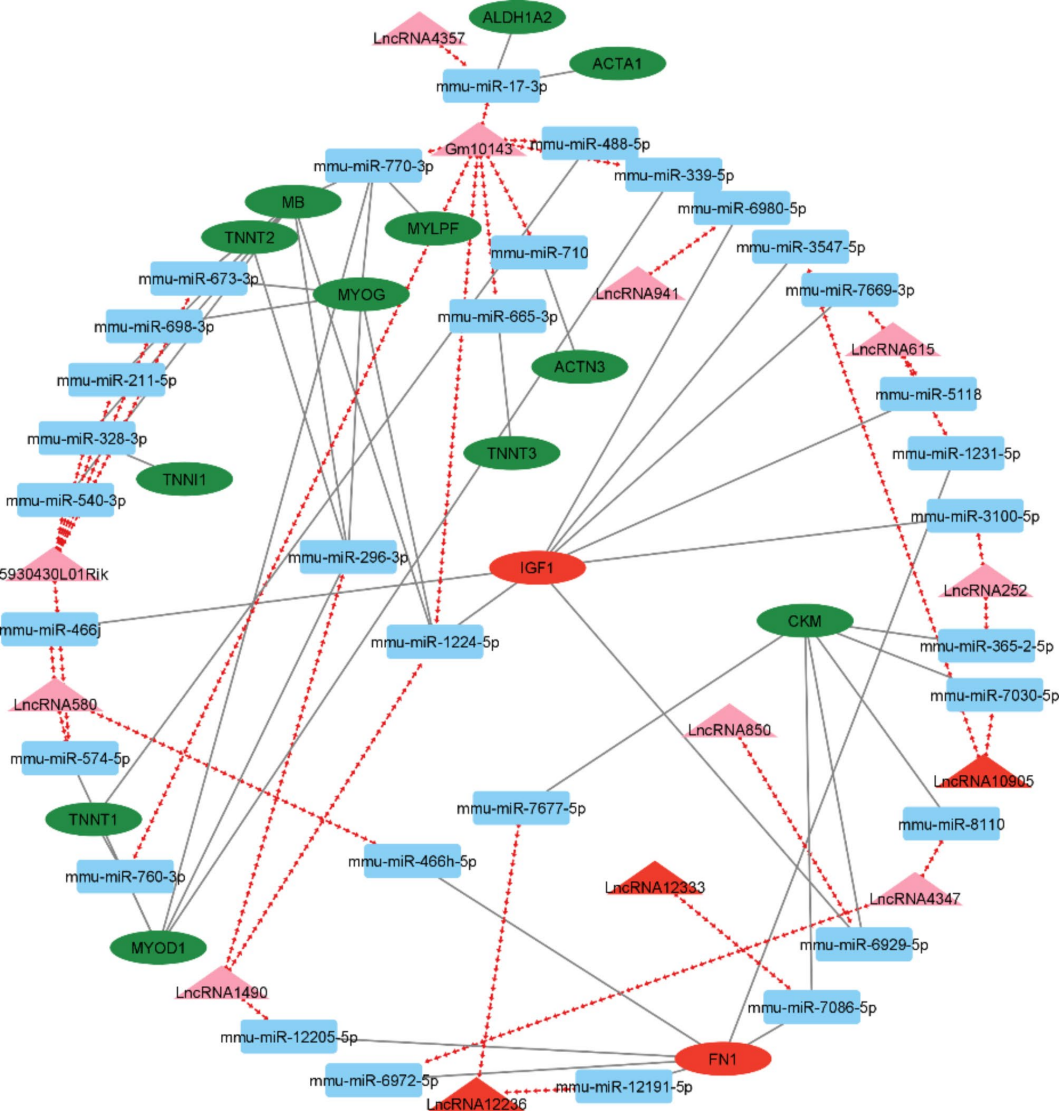
The ceRNA network of lncRNA–miRNA–mRNA interactions. Rectangles, circles, and triangles represent miRNAs, mRNAs, and lncRNAs, respectively. Solid gray lines indicate interactions between miRNA-mRNA, while red arrows depict miRNA-lncRNA interactions. In this network, the red and green color for genes and lncRNA corresponds to down and upregulation, respectively.

 The concept of the competing endogenous RNAs (ceRNA) network involves a regulatory system of RNA molecules that interact with each other, mutually influencing their post-transcriptional regulation by miRNA. The ceRNA networks provide another type of function for mRNAs, which link non-coding RNAs such as miRNA, lncRNA, pseudogenes, and circular RNAs^17^. According to the ceRNA theory, we used the shared miRNA as a junction to construct a lncRNA–miRNA–mRNA, a ceRNA network consisting of 62 nods and 86 interactions, including 34 miRNAs, 14 lncRNAs, and 14 mRNAs (Fig. 3).

### Literature analysis

**Fig. 4:**
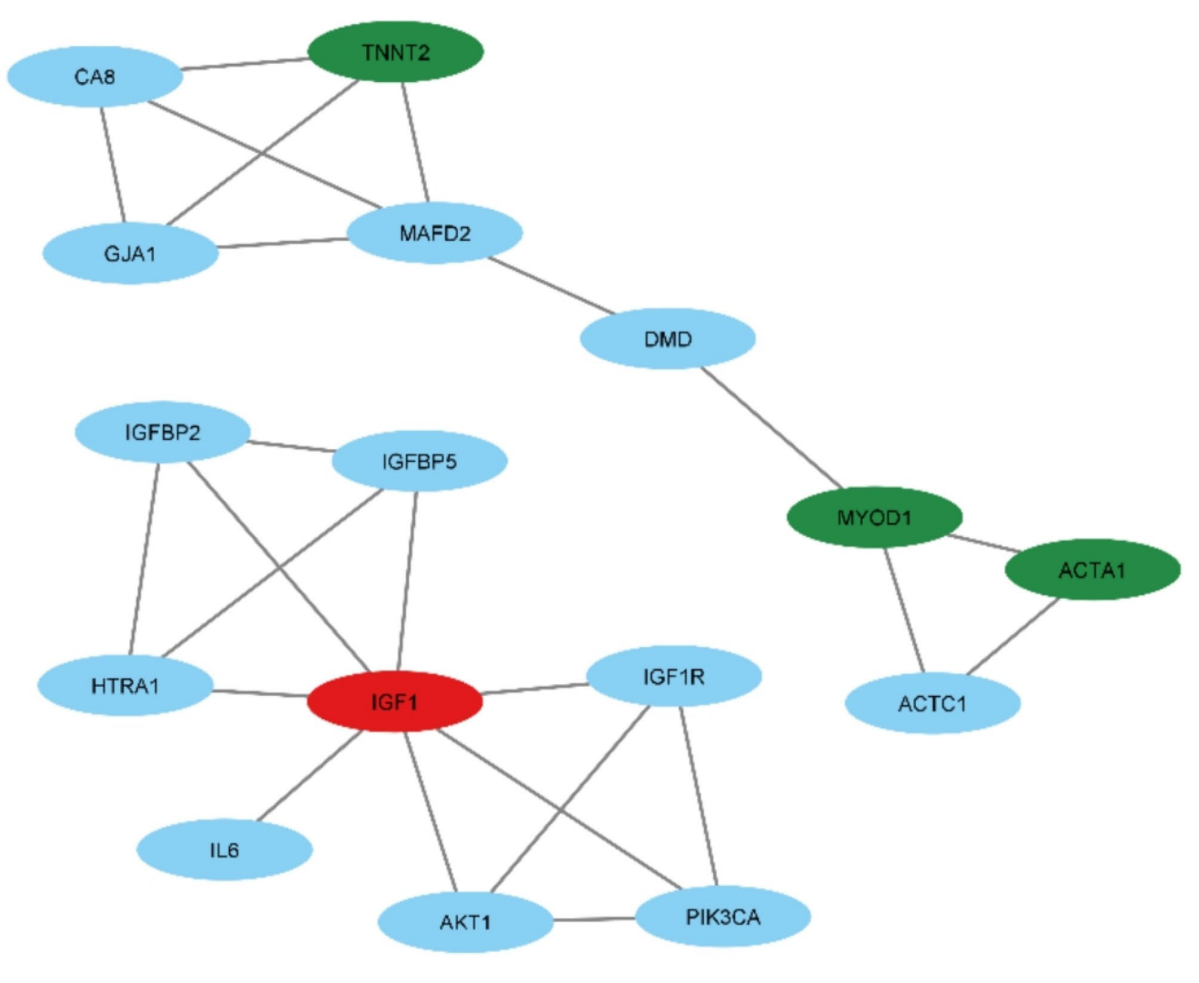
Interactions between hub genes and other genes in the context of DMD disease. In this network, the red and green color for genes and lncRNA corresponds to down and upregulation, respectively. The blue color shows the genes found based on Agilent Literature Search software.

 The software tool known as Agilent Literature Search serves as a valuable asset in the realm of biomedical text mining and biological network analyses. Its primary function is to conduct literature searches for molecules or topics of interest and then utilize this information to generate networks based on the relationships between them^18^. We have uncovered significant connections and interactions within the network based on the analysis performed using Agilent Literature Search. Our findings revealed that the gene *TNNT2* is connected to *GIA1*, *MAFD2*, and *CA8*. Furthermore, we have observed interactions between *MYOD1*, Dystrophin, and *ACTC1*, suggesting regulatory relationships or functional links between these genes. Additionally, our analysis has highlighted the involvement of *IGF1* in interactions with several other genes and proteins. Specifically, *IGF1* exhibits interactions with *IGFBP2*,* IGFBP5*, *HTRA1*, *IGF1R*,* IL6*, *AKT1*, and *PIK3CA* (Fig. 4). These interactions confirm/suggest the potential roles of *IGF1* in various biological processes, such as growth regulation, signaling pathways, and cellular responses.

**Fig. 5:**
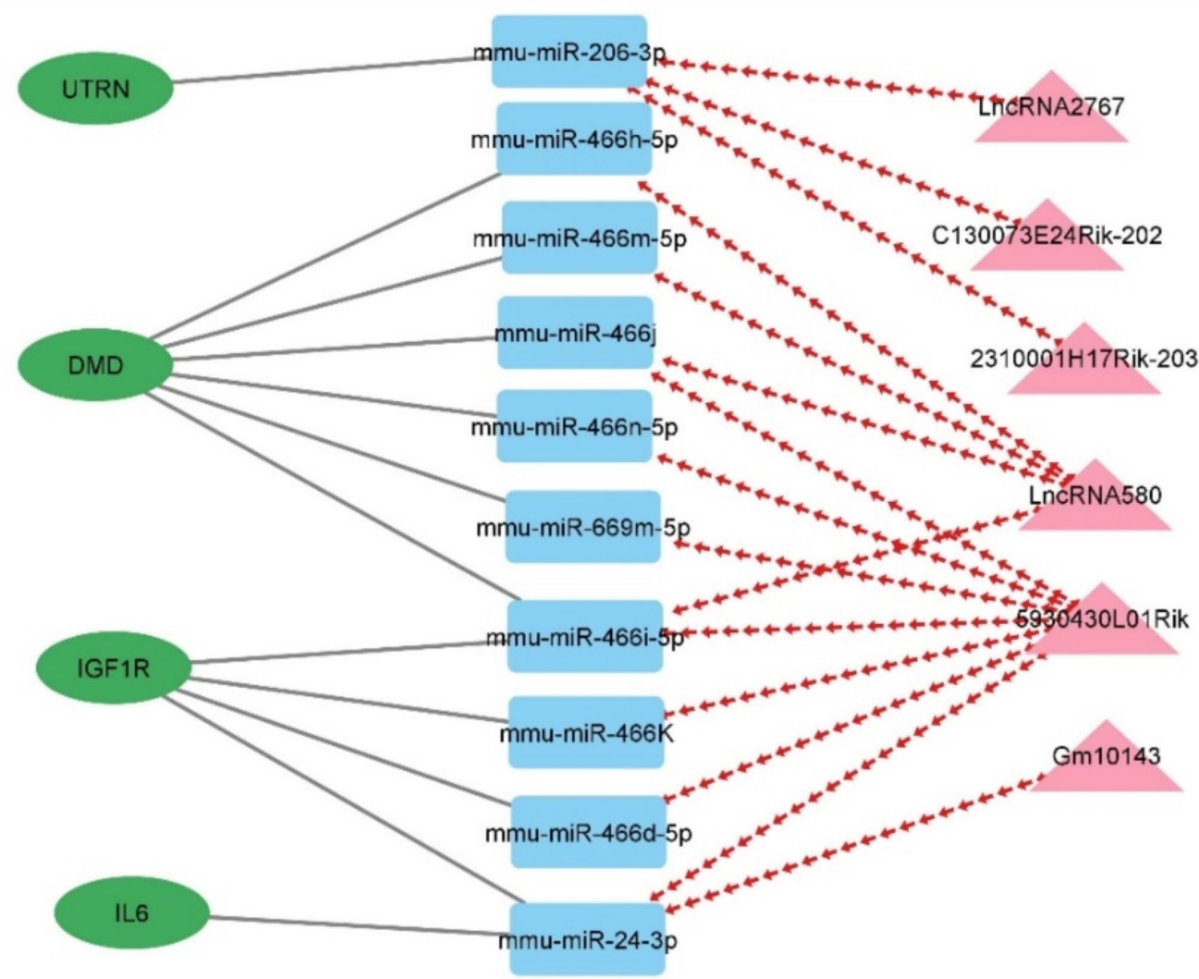
Interaction of miRNAs common between lncRNA and identified genes. Rectangles, circles, and triangles represent miRNAs, mRNAs, and lncRNAs, respectively. Solid gray lines indicate interactions between miRNA-mRNA, while red arrows depict miRNA-lncRNA interactions.

Our study focused on identifying interactions between 34 miRNAs that are shared between lncRNAs and mRNA (Fig. 2). To accomplish this, we utilized two software tools, miRCARTA and miRNet, widely used for miRNA target prediction and analysis. We successfully identified additional genes that interact with the miRNAs of interest by employing these software tools. Our analysis revealed that four important genes holding critical roles in the context of DMD disease, namely *Dystrophin*, *UTRN*, *IGF1R*, and *IL6*, were enriched with aforementioned miRNAs (Fig. 5).

## Discussion

In the current study, we created a network connecting variably expressed lncRNAs identified in DMD/mdx myoblasts by RNA-seq with predicted miRNAs and mRNAs previously identified by Gosselin et al.^16^ to understand the role of lncRNAs in DMD disease. In the present study, 554 differentially expressed lncRNAs were identified, and the top 10 upregulated and downregulated lncRNAs were selected for in-depth analysis (Fig. 1). Additionally, we included in the study a dataset consisting of 170 mRNAs identified by Gosselin et al.^16^ to investigate the links between lncRNAs and mRNAs.

This investigation identified a lncRNA called Gm10143, which exhibited significant overexpression in DMD/mdx myoblasts. This particular lncRNA was found to be associated with the regulation of several important genes in skeletal muscle development, function, and disease, namely *CTA1*,* ACTN3*,* ALDH1A2*,* MB*,* MYLPF*,* MYOD1*,* MYOG*,* TNNT1*, and *TNNT3* (Table 2). Notably, these genes demonstrated an upregulation pattern in response to the overexpression of Gm10143 within the context of this study. The findings suggest that Gm10143 might play a vital role in modulating the expression of aforementioned genes^2,19–21^. The interaction between Gm10143 and specific miRNAs (mmu-miR-710, mmu-miR-665-3p, mmu-miR-1224-5p, mmu-miR-760-3p, mmu-miR-770-3p, mmu-miR-488-5p, and mmu-miR-17-3p) could potentially explain the observed regulatory effects on above-mentioned target genes. Our study predicts that Gm10143 might regulate miR-710, which can regulate *ACTN3* gene expression. Hogarth et al.^22^ show that *ACTN3* (*R577X*) reduces muscle strength in young patients with DMD. In addition, this lncRNA might control *MYOD1* expression regulated by some miRNAs, namely mmu-miR-339-5p, mmu-miR-760-3p, mmu-miR-760-3p, and mmu-miR-770-3p (Fig. 3). *MYOD1* plays a crucial role in muscle development and regeneration, and its absence in the mdx/MYOD^−/−^ mouse model leads to increased disease severity. However, *MYOD1* is not directly involved in the pathogenesis of DMD in humans^23^. According to literature analysis, *MYOD1*-converted urine-derived cells are a novel DMD muscle cell model used to evaluate exon-skipping drugs targeting *Dystrophin* exons, including 44, 50, 51, and 55, which shows that there is a link between the *MYOD1* gene and *Dystrophin*, enabling *MYOD1* to be used as a tool to study DMD and evaluate potential treatments^24^, including the identified lncRNA. DMD patient-derived myoblasts exhibit reduced *MYOD1* expression levels compared to myoblasts from healthy individuals. This decline in *MYOD1* within DMD-myoblasts expression is linked to hindered myotube formation. The genetic intervention, such as introducing the complete human *Dystrophin* gene, reverses the transcriptional profile and upregulation of *MYOD1* expression in corrected DMD-myoblasts^25^. Using lncRNA that, by sponging, reduces miRNA activity may potentially increase *MYOD1* protein level.

5930430L01Rik, an upregulated lncRNA, is suggested to be linked to genes such as MB, TNNI1, TNNT2, and *MYOD1*. These genes are upregulated in DMD/mdx myoblasts compared to WT^16,26^. Available literature data indicate that *TNNI1* was identified as a marker for skeletal muscle cells and was found to be expressed at high levels in the myocytes differentiated from tonsil-derived mesenchymal stem cells^26^. Our analysis suggests that 5930430L01Rik may regulate the activity of this gene by sponging/regulating the mmu-miR-328-3p molecule.

Myogenesis refers to the formation of muscle tissue, and understanding the control of this process may be necessary to treat DMD^27^. Gosselin et al.^16^ identified MYOG as an upregulated gene in DMD/mdx myoblasts compared to WT cells. This upregulation is notably influenced by a network of miRNAs, including mmu-miR-673-3p, mmu-miR-698-3p, mmu-miR-1224-5p, mmu-miR-770-3p, and mmu-miR-296-3p. Interestingly, these miRNAs are controlled by three identified in our analysis lncRNAs upregulated in DMD/mdx myoblasts, namely 5930430L01Rik, Gm10143, and LncRNA1490. Based on the observed overexpression of lncRNAs and increased expression of *MYOG* in DMD/mdx myoblasts, it can be assumed that there is a positive correlation between the lncRNAs mentioned above and the *MYOG* transcription factor.

*IGF1* is a gene that exhibits decreased expression in DMD/mdx myoblasts compared to WT cells^16^ and plays a significant role in the development, maintenance, and regeneration of skeletal muscles^28^. According to our analysis, this gene can be regulated by several miRNAs, including mmu-miR-3547-5p, mmu-miR-1224-5p, mmu-miR-3100-5p, mmu-miR-466j, mmu-miR-7669-3p, mmu-miR-6929-5p, mmu-miR-5118, and mmu-miR-6980-5p. We observed a regulatory relationship involving the aforementioned miRNAs and several lncRNAs. Namely, lncRNA10905, that was downregulated, and lncRNA1490, lncRNA252, lncRNA580, lncRNA615, lncRNA850, and lncRNA941 that exhibited an overexpression pattern. *IGF1* increases the proliferative capacity of muscle satellite cells and stimulates their proliferation and myogenic differentiation. Mechanical loading also affects the production of *IGF1* by skeletal muscle, and low *IGF1* levels are associated with low handgrip strength and poor physical performance. *IGF1* is potentially useful in managing DMD and muscle atrophy and promotes neurite development^28^. IGF2 is a particularly noteworthy regulator of cell growth, survival, migration, and differentiation in the IGF family. An increase in IGF2R expression was documented in dystrophic skeletal muscle in both humans with DMD and mdx mice. Inhibiting IGF2R activity with antibodies showed noticeable consequences in dystrophic mdx mice, triggering muscle regeneration and strength restoration. This discovery serves as a promising starting point for exploring and developing novel biological therapeutics designed to target DMD^29,30^, and the lncRNAs and miRNAs we identified can potentially support this process.

A protein called cardiac troponin T, which is involved in muscle contraction, is produced by the *TNNT2* gene. Bakay et al.^31^ found a 10-fold upregulation in *TNNT2* levels in DMD. In dystrophin-deficient human muscle, increased *TNNT2* expression was observed in activated myoblasts^31^. Missplicing of the *TNNT2* gene has been observed in skeletal muscle in myotonic dystrophy type 1 (DM1). In DM1, the *TNNT2* gene shows the same aberrant splicing pattern as observed in cardiac muscle^32^. The activity of this gene, the expression of which was increased in the studies of Gosselin et al.^16^, may be affected by two lncRNAs, namely 5930430L01Rik and lncRNA1490, which were observed to be overexpressed in our study. We suppose that lncRNAs mentioned above regulate *TNNT2* directly or by controlling mmu-miR-673-3p and mmu-miR-296-3p activity.

*FN1*, a downregulated gene identified in DMD/mdx myoblasts^16^, is a molecule that plays a vital role in the development of fibrotic disease by regulating the deposition and assembly of several extracellular matrix components, including collagens, as well as *TGF-β1* factor. *FN1* gene expression may be regulated by some lncRNAs identified in our analysis, namely lncRNA12236, lncRNA12333, lncRNA1490, lncRNA4357, lncRNA580, and lncRNA615. Zanotti et al.^33^ found that pirfenidone treatment significantly downregulated fibronectin expression in muscle-derived fibroblasts from DMD patients, indicating its potential use in treating fibrosis in dystrophic skeletal muscle. Another study identified *COL1A2*,* FBN1*, and *FN1* as potential biomarkers for DMD; however, in that investigation, *FN1* was upregulated in DMD/mdx myoblasts^34^.

Our study highlights the correlation and the biological effect of miRNAs that might contribute to some extent to expanding our knowledge about the effects of certain miRNAs on DMD. Based on bioinformatic analysis, we identified several miRNAs common to lncRNA and mRNA (Fig. 3). Among them, miR-206, miR-466, miR-669, and miR-24 were particularly noteworthy. MiR-206, belonging to the muscle-specific miRNAs (myomiRs) category, has the potential as a biomarker to track muscle pathology and assess disease severity in DMD patients. It is well-known that the expression of miR-206 is elevated in individuals affected by DMD^35–37^. Moreover, this miRNA is linked to the utrophin gene (*UTRN)*, the expression of which is upregulated in DMD disease^38–40^. The *UTRN* gene, responsible for encoding the utrophin protein, has gained attention due to its potential role in the treatment of functional dystrophin deficiency in DMD. Studies have shown a direct interaction between the homeobox protein engriled-1 (EN1) and the *UTRN* promoter. That inhibition of EN1 gene expression increases utrophin mRNA levels, suggesting that increasing *UTRN* expression may serve as a compensatory mechanism for dystrophin deficiency in DMD^41–43^. Our findings suggest a potential regulatory mechanism involving three overexpressed lncRNAs 2310001H17Rik-203, C130073E24Rik-202, and lncRNA2767. These lncRNAs seem to function as sponges for miR-206-3p, resulting in an increased expression of the UTRN gene in DMD/mdx myoblasts. However, for a more comprehensive understanding, experimental validation is necessary.

Moreover, we have identified a significant connection between miR-466i-5p, miR-466n-5p, miR-466d-5p, miR-466 h-5p, miR-466j, and miR-466k, and two important genes, namely *Dystrophin* and *IGF1*. This miRNA family is regulated by two upregulated lncRNAs identified in our analysis, such as 5930430L01Rik and lncRNA580. Furthermore, we observed that 5930430L01Rik regulates miR-669 m-5p, which in turn controls *Dystrophin* gene expression. MiR-669 m-5p has been shown to play a vital role in ensuring proper muscle structure and functionality, especially in mice with dystrophy and dilated cardiomyopathy. Additionally, miR-669a treatment led to several positive effects, including improved sarcomere organization, reduced ventricular atrial natriuretic peptide levels, and improved gene/miRNA profile associated with dilated cardiomyopathy^44^.

## Materials and methods

### Data collection

**Fig. 6:**
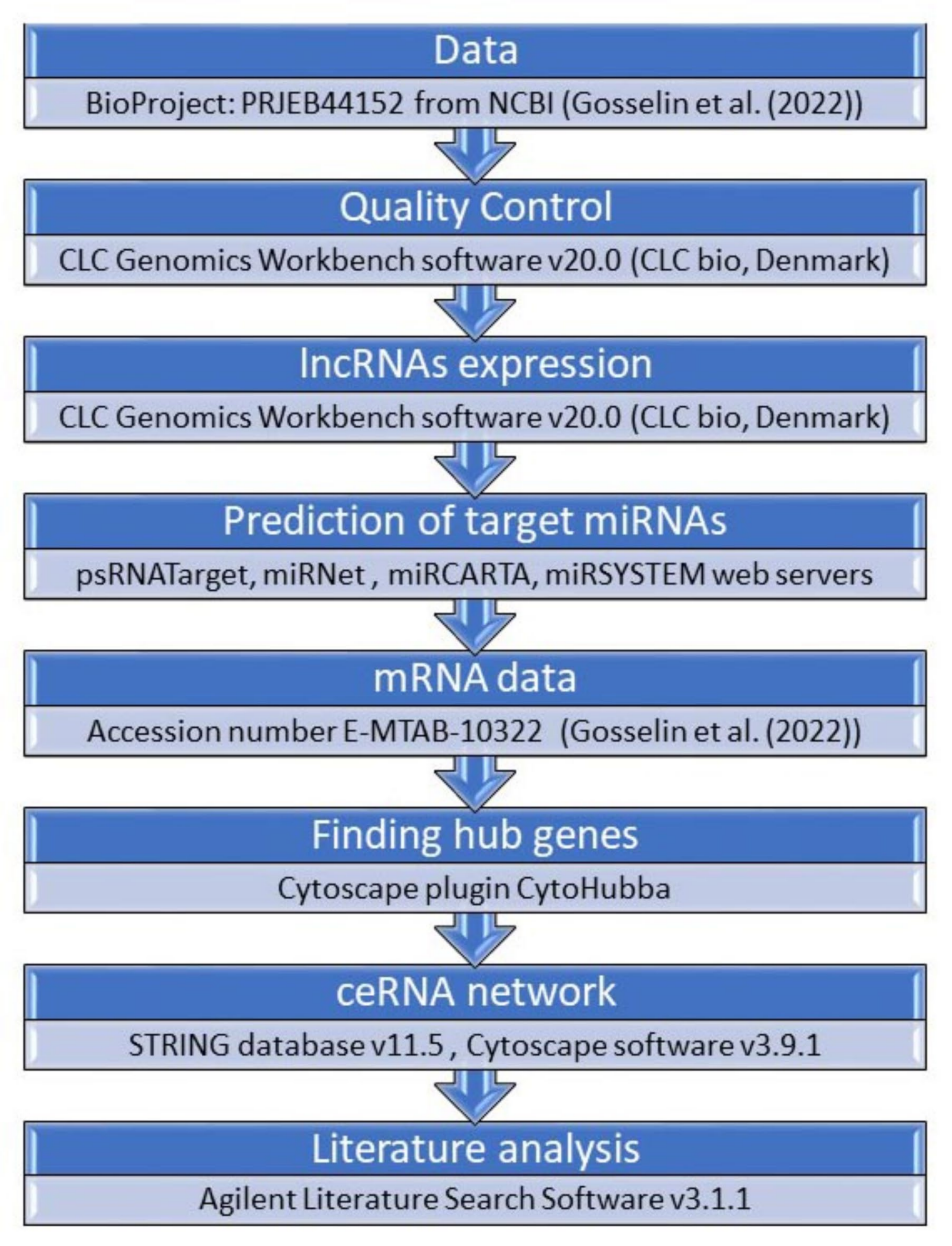
Workflow showing the basic steps of analyzing lncRNA expression, discovering new lncRNAs, and identifying their interactions with mRNA. The study focused on quality control and identification of long non-coding RNAs (lncRNAs) in *Mus musculus* using CLC Genomics Workbench 20.0 V software. Sequencing data underwent rigorous filtering, including quality score limits, ambiguity thresholds, and adapter trimming. Identification of putative lncRNAs involved removing protein-coding sequences and filtering against undesirable genomic regions. De novo assembly was performed, and BLAST searches against protein databases and Pfam were executed to assess coding potential. Coding Potential Calculator (CPC2) and Ensemble searches further refined candidates. Differentially expressed lncRNAs in DMD/mdx versus WT myoblasts were identified through RNA-seq analysis, employing empirical testing and Volcano plots with specific fold change and p-value criteria. The preprocessing steps ensured high-quality, error-minimized data for subsequent analyses.

 The expression profiles of the primary myoblasts isolated from gastrocnemius muscle of three 8-week-old male DMD/mdx mice (lacking the full-length *Dystrophin* transcript) and from the muscle of three WT control mice (BioProject: PRJEB44152) deposited by Gosselin et al.^16^ were obtained from NCBI (http://www.ncbi.nlm.nih.gov/sra). Authors investigated genetic changes in primary myoblasts from mice lacking the full-length dystrophin transcript, a critical factor in Duchenne muscular dystrophy. Using RNA sequencing on 8-week-old male DMDmdx and control mice, the research identifies altered gene expression, shedding light on potential intervention targets. The analysis reveals 170 significantly altered genes, including ex. Myod1 and essential genes controlled by it and associated with muscle development and function. The total RNA extraction, RNA-Seq paired-end sequencing (Illumina HiSeq 2500), and raw data statistics were described by Gosselin et al.^16^. The workflow of our in silico analysis is presented in Fig. 6.

### Quality control of raw sequencing data analysis

Identifying lncRNAs in the *Mus musculus* genome was possible using the transcript detection plug-in available in the CLC Genomics Workbench 20.0 V software (CLC bio, Denmark). With the aid of extensive gap mapping, 390,859,276 reads in FASTA format from the NCBI database of six RNA-Seq libraries were mapped to the genomic reference, identifying new transcripts. This approach has proven an effective tool for discovering lncRNAs in the genome. Raw reads were filtered by a quality score limit of 0.05 and a maximum number of 2 ambiguities, and adapter/index trimmed and removed sequences that were less than 15 base pairs.

Identification of putative long non-coding transcripts involves the removal of sequences that could encode the protein. Then, various filters were systematically applied to identify lncRNAs. Subsequently, we aligned the data that was trimmed to the *Mus musculus* reference genome (GCF_000001635.sw41Y3mB. 27_GRCm39_genomic.gbff.gz) received from NCBI (https://www.ncbi.nlm.nih.gov). To refine the data and enhance its accuracy, we performed mappings on different tracks, eliminating mapped sequences that overlapped with undesirable regions such as genes, coding sequences (CDS), mRNA, and other genome tracks. However, non-coding regions were preserved in the analysis. After completing the filtering process, we proceeded with *de novo* assembly. *De novo* assembly algorithm of CLC software was used to create a contig list from previously filtered reads using a mismatch cost = 2, insertion cost = 3, deletion cost = 3, length fraction = 0.8, similarity fraction = 0.8, and a minimum contig length = 250 (We opted for a minimum contig length of 250 to enhance confidence and accuracy in our selections).

Any possible similarity with other known proteins was found using the BLASTx algorithm against human and animal protein databases from UniProt (https://www.uniprot.org/) and NCBI (https://www.ncbi.nlm.nih.gov/protein/), as well as the Pfam database (https://pfam.xfam.org). All possible six frames were prepared for all selected sequences. Then, the translated sequences were subjected to a domain search to identify any putative conserved protein domains through the Pfam v27.0 database.

Coding Potential Calculator (CPC2) tool (http://cpc.cbi.pku.edu.cn) was used to check for other potential coding regions. CPC2 is a Support Vector Machine-based classifier that can assess a transcript’s protein-coding potential based on six biologically meaningful sequence features. To identify both known and unknown lncRNAs, we performed a BLAST search against the Mus_musculus.GRCm39. The ncrna.fa.gz file is available on the Ensemble website (https://www.ensembl.org). These candidates were refined through extensive filtration and assembly steps, which helped us obtain a more precise dataset for further research studies. The preprocessing steps we performed ensured that the data we obtained and utilized for our analysis was of high quality and had minimal errors or discrepancies.

### Analysis of differentially expressed lncRNAs

 We identified differentially expressed lncRNAs by mapping filtered reads to RNA-seq results. Parameters considered included mapping settings based on the default CLC software (considering a mismatch cost = 2, insertion cost = 3, deletion cost = 3, length fraction = 0.8, and a similarity fraction = 0.8.). Expression values were determined as Reads Per Kilobase of transcript per Million mapped reads (RPKM). To identify lncRNAs highly regulated in DMD/mdx in comparison to WT myoblasts, the empirical analysis for different lncRNA expression was used. This test gathers experimental data and compares all samples to each other. An empirical analysis involves examining expression data to determine whether there is evidence that counts for a transcript or exon are significantly different across experimental conditions^45^. A Volcano plot was used to select and extract the most differentially expressed transcripts with fold change > 2 and p-value < 0.05, compared to the WT control group.

### Prediction of miRNAs for lncRNA and mRNA-target interactions

 For prediction interaction between lncRNA and miRNA, we used the psRNATarget web server (https://www.zhaolab.org/psRNATarget/analysis). The psRNATarget web server is a widely used tool designed to predict potential interactions between miRNAs and target RNA molecules, including lncRNAs. This server incorporates sophisticated algorithms and databases to enhance the accuracy of the predictions^46^. Three algorithms, such as miRNet47(https://www.mirnet.ca/), miRCARTA (https://mircarta.cs.uni-saarland.de), and miRSYSTEM (http://mirsystem.cgm.ntu.edu.tw) were used for miRNA-mRNA target gene prediction using the default setting. Target miRNAs that overlapped with lncRNAs and mRNAs were used to construct a competitive endogenous RNA (ceRNA) network.

### LncRNA-miRNA-mRNA and PPI network construction

 The PPI network of differentially expressed genes included in the ceRNA network was created using the STRING database version 11.5 (https://string-db.org/). Interactions with a validation score > 0.4 were defined as significant, and validated networks were visualized in Cytoscape (v3.9.1). The lncRNA-miRNA-mRNA network was constructed based on the theory that ceRNAs bind miRNAs competitively through the same miRNA response elements (MREs). Finally, the lncRNA-miRNA-mRNA network was structured by combining all the co-expressed competitive triplets identified as described above and visualized Cytoscape software (v3.9.1).

### Network analysis

 The powerful CytoHubba plugin for Cytoscape software, a widespread network analysis and visualization software platform, was used^48^. It provides a range of algorithms designed to identify important nodes or hubs within a network and offers several ranking methods that assess the significance of nodes based on their connectivity patterns, including degree centrality—measures the number of connections a node has in a network; betweenness centrality—identifies nodes that act as bridges or intermediaries between other nodes in the network; closeness centrality—evaluates how quickly information can spread from a given node to all other nodes in the network. Additionally, CytoHubba includes other algorithms like differential multi-network centrality (DMNC) and maximal clique centrality (MCC) for finding hub genes. DMNC considers multiple networks and their activities to identify critical nodes crucial in distinguishing between states or conditions. MCC identifies nodes within maximal cliques that are fully connected subgraphs and prioritizes them as potential hub nodes^48^. Utilizing these algorithms, we identified key nodes/proteins that play an essential role in the identified relevance network.

### Literature analysis

 Agilent Literature Search software (v3.1.1) as a Cytoscape plug-in is an automated meta-search tool that helps search and extract associations between genes/proteins of interest by querying multiple text-based search engines and preparing a network view of gene/protein associations. In our analysis, false-positive interaction information was removed from search results, then gene/protein interaction relationships were loaded in Cytoscape 3.9.1 and visualized.

## Conclusions

Identification of differentially expressed lncRNA and analysis of their interactions in the ceRNA network in DMD/mdx myoblast cells revealed the ten most upregulated and downregulated lncRNAs that show the potential to indirectly control DMD-related gene expression, possibly contributing to the regulation of molecular mechanisms determining the occurrence of DMD disease. The results of this study not only highlight the presence of lncRNAs in DMD/mdx myoblast cell cultures - a model of Duchenne muscular dystrophy- and serve as a valuable reference for understanding the involvement of hub genes in the regulation of DMD. These results shed light on the complex regulatory network involving lncRNAs, miRNAs, and genes involved in the pathogenesis of DMD, providing a basis for further research and potential therapeutic interventions.

## Electronic supplementary material

Below is the link to the electronic supplementary material.

Supplementary Material 1

Supplementary Material 2

## Data Availability

If more data is required than presented in the article, the corresponding author will make these data available upon request. If more data is needed than presented in the article, the corresponding author will make these data available upon request. The analysis described in this article uses raw data ERR5671405, ERR5671406, ERR5671407, ERR5671408, ERR5671409, and ERR5671410 publicly available at http://www.ncbi.nlm.nih.gov/sra.

## References

[1] Flores-Concha, M. & Oñate, Á. A. Long non-coding RNAs in the regulation of the immune response and trained immunity. *Front. Genet. ***11**, 718 (2020). 32793280 10.3389/fgene.2020.00718 PMC7393263

[2] Wang, W. et al. Biological function of long non-coding RNA (LncRNA) xist. *Front. cell. Dev. Biol. ***9**, 645647 (2021). 34178980 10.3389/fcell.2021.645647 PMC8222981

[3] Fang, Y. et al. Recent advances on the roles of LncRNAs in cardiovascular disease. *J. Cell. Mol. Med. ***24**, 12246–12257 (2020). 32969576 10.1111/jcmm.15880 PMC7686979

[4] Hu, B., Chen, W., Zhong, Y. & Tuo, Q. The role of lncRNA-mediated pyroptosis in cardiovascular diseases. *Front. Cardiovasc. Med. ***10**, 1217985 (2023). 37396588 10.3389/fcvm.2023.1217985 PMC10313127

[5] Ko, N. Y., Chen, L. R. & Chen, K. H. The role of micro RNA and long-non-coding RNA in osteoporosis. *Int. J. Mol. Sci. ***21**, 4886 (2020). 32664424 10.3390/ijms21144886 PMC7402348

[6] Simion, V., Haemmig, S. & Feinberg, M. W. LncRNAs in vascular biology and disease. *Vascul. Pharmacol. ***114**, 145–156. 10.1016/j.vph.2018.01.003 (2019). 29425892 10.1016/j.vph.2018.01.003 PMC6078824

[7] Hua, X. et al. Multi-level transcriptome sequencing identifies COL1A1 as a candidate marker in human heart failure progression. *BMC Med. ***18**, 1–16 (2020). 31902369 10.1186/s12916-019-1469-4 PMC6943904

[8] Omura, J. et al. Identification of long noncoding RNA H19 as a new biomarker and therapeutic target in right ventricular failure in pulmonary arterial hypertension. *Circulation ***142**, 1464–1484 (2020). 32698630 10.1161/CIRCULATIONAHA.120.047626

[9] Bitarafan, S. et al. Association of increased levels of lncRNA H19 in PBMCs with risk of coronary artery disease. *Cell. J. (Yakhteh) ***20**, 564 (2019). 10.22074/cellj.2019.5544 PMC6099137 30124004

[10] Chen, W. et al. Role of lncRNA Has2os in skeletal muscle differentiation and regeneration. *Cells ***11**, 3497 (2022). 36359891 10.3390/cells11213497 PMC9655701

[11] Qi, X. et al. LncRNAs are regulated by chromatin states and affect the skeletal muscle cell differentiation. *Cell Prolif. ***53**, e12879 (2020). 32770602 10.1111/cpr.12879 PMC7507427

[12] Li, Z. et al. Integrated analysis of long non-coding RNAs (LncRNAs) and mRNA expression profiles reveals the potential role of LncRNAs in skeletal muscle development of the chicken. *Front. Physiol. ***7**, 687 (2017). 28119630 10.3389/fphys.2016.00687 PMC5220077

[13] Alessio, E. et al. Single cell analysis reveals the involvement of the long non-coding RNA Pvt1 in the modulation of muscle atrophy and mitochondrial network. *Nucleic Acids Res. ***47**, 1653–1670 (2019). 30649422 10.1093/nar/gkz007 PMC6393313

[14] Shastry, A. et al. Matrilineal analysis of mutations in the DMD gene in a multigenerational south Indian cohort using DMD gene panel sequencing. *Mol. Genet. Genom. Med. ***9**, e1633 (2021). 10.1002/mgg3.1633 PMC8172192 33960727

[15] Gargaun, E. et al. The lncRNA 44s2 study applicability to the design of 45–55 exon skipping therapeutic strategy for DMD. *Biomedicines ***9**, 219 (2021). 33672764 10.3390/biomedicines9020219 PMC7924625

[16] Gosselin, M. R. et al. Loss of full-length dystrophin expression results in major cell-autonomous abnormalities in proliferating myoblasts. *Elife ***11**, e75521 (2022). 36164827 10.7554/eLife.75521 PMC9514850

[17] Zhang, Z. K. et al. Long noncoding RNA lncMUMA reverses established skeletal muscle atrophy following mechanical unloading. *Mol. Ther. ***26**, 2669–2680 (2018). 30415659 10.1016/j.ymthe.2018.09.014 PMC6225098

[18] Bernabo, N., Ordinelli, A., Di Agostino, R., Mattioli, M. & Barboni, B. Network analyses of sperm–egg recognition and binding: ready to Rethink Fertility mechanisms? *OMICS: J. Integr. Biol. ***18**, 740–753 (2014). 10.1089/omi.2014.0128 PMC4253145 25454512

[19] Novelli, G. et al. Polymerase chain reaction in the detection of mRNA transcripts from the slow skeletal troponin T (TNNT1) gene in myotonic dystrophy and normal muscle. *Cell. Biochem. Funct. Cell. Biochem. Modul. Act. Agents Dis. ***10**, 251–256 (1992). 10.1002/cbf.290100407 1473264

[20] Florczuk, P., Maciej, M., Matuszewski, A. & Gruszczyńska, J. Myogenic regulatory factors in myogenesis and regeneration of skeletal muscle. *World Sci. News ***2**, 120–126 (2017).

[21] Rihani, L., Liesche-Starnecker, F. & Schlegel, J. Human skeletal muscle satellite cells co-express aldehyde dehydrogenase isoforms Aldh1a1 and Aldh1A3 (2021).

[22] Hogarth, M. W. et al. Evidence for ACTN3 as a genetic modifier of Duchenne muscular dystrophy. *Nat. Commun. ***8**, 14143 (2017). 28139640 10.1038/ncomms14143 PMC5290331

[23] Yucel, N., Chang, A. C., Day, J. W., Rosenthal, N. & Blau, H. M. Humanizing the mdx mouse model of DMD: the long and the short of it. *NPJ Regener. Med. ***3**, 4 (2018). 10.1038/s41536-018-0045-4 PMC5816599 29479480

[24] Takizawa, H. et al. Modelling Duchenne muscular dystrophy in MYOD1-converted urine-derived cells treated with 3-deazaneplanocin A hydrochloride. *Sci. Rep. ***9**, 3807 (2019). 30846748 10.1038/s41598-019-40421-z PMC6405839

[25] Choi, I. Y. et al. Concordant but varied phenotypes among Duchenne muscular dystrophy patient-specific myoblasts derived using a human iPSC-based model. *Cell. Rep. ***15**, 2301–2312 (2016). 27239027 10.1016/j.celrep.2016.05.016

[26] Choi, Y. et al. Biochemical and functional characterization of skeletal muscle cells differentiated from tonsil-derived mesenchymal stem cells. *Muscle Nerve* (2023). 10.1002/mus.27847 37243484

[27] Farini, A., Razini, P., Erratico, S., Torrente, Y. & Meregalli, M. Cell based therapy for Duchenne muscular dystrophy. *J. Cell. Physiol. ***221**, 526–534 (2009). 19688776 10.1002/jcp.21895

[28] Ahmad, S. S., Ahmad, K., Lee, E. J., Lee, Y. H. & Choi, I. Implications of insulin-like growth factor-1 in skeletal muscle and various diseases. *Cells ***9**, 1773 (2020). 32722232 10.3390/cells9081773 PMC7465464

[29] Bella, P. et al. Blockade of IGF2R improves muscle regeneration and ameliorates Duchenne muscular dystrophy. *EMBO Mol. Med. ***12**, e11019 (2020). 31793167 10.15252/emmm.201911019 PMC6949491

[30] Patel, K., Macharia, R. & Amthor, H. Molecular mechanisms involving IGF-1 and myostatin to induce muscle hypertrophy as a therapeutic strategy for Duchenne muscular dystrophy. *Acta Myol.: Myopathies Cardiomyopathies: Off. J. Mediterranean Soc. Myol. ***24**, 230–241 (2005). 16629058

[31] Bakay, M., Zhao, P., Chen, J. & Hoffman, E. P. A web-accessible complete transcriptome of normal human and DMD muscle. *Neuromuscul. Disord. ***12**, S125–S141 (2002). 12206807 10.1016/s0960-8966(02)00093-7

[32] Bosè, F. et al. TNNT2 missplicing in skeletal muscle as a cardiac biomarker in myotonic dystrophy type 1 but not in myotonic dystrophy type 2. *Front. Neurol. ***10**, 992 (2019). 31611837 10.3389/fneur.2019.00992 PMC6776629

[33] Zanotti, S. et al. Anti-fibrotic effect of pirfenidone in muscle derived-fibroblasts from Duchenne muscular dystrophy patients. *Life Sci. ***145**, 127–136 (2016). 26679108 10.1016/j.lfs.2015.12.015

[34] Li, N., Zhikai, X., Li, Z., Zhang, Z. & Song, Y. Identification of hub genes and therapeutic siRNAs to develop novel adjunctive therapy for Duchenne muscular dystrophy (2022). 10.1186/s12891-024-07206-6 PMC11102231 38762732

[35] Almeida-Becerril, T. et al. Natural history of circulating miRNAs in Duchenne disease: Association with muscle injury and metabolic parameters. *Acta Neurol. Scand. ***146**, 512–524 (2022). 36000352 10.1111/ane.13673

[36] García-Giménez, J. L. et al. Identification of circulating miRNAs differentially expressed in patients with Limb-girdle, Duchenne or facioscapulohumeral muscular dystrophies. *Orphanet J. Rare Dis. ***17**, 450 (2022). 36575500 10.1186/s13023-022-02603-3 PMC9793535

[37] Meng, Q., Zhang, J., Zhong, J., Zeng, D. & Lan, D. Novel miRNA biomarkers for patients with Duchenne muscular dystrophy. *Front. Neurol. ***13**, 921785 (2022). 35873767 10.3389/fneur.2022.921785 PMC9298557

[38] Arechavala-Gomeza, V. et al. Immunohistological intensity measurements as a tool to assess sarcolemma‐associated protein expression. *Neuropathol. Appl. Neurobiol. ***36**, 265–274 (2010). 20002311 10.1111/j.1365-2990.2009.01056.x

[39] Helliwell, T., Morris, G. & Davies, K. The dystrophin-related protein, utrophin, is expressed on the sarcolemma of regenerating human skeletal muscle fibres in dystrophies and inflammatory myopathies. *Neuromuscul. Disord. ***2**, 177–184 (1992). 1483043 10.1016/0960-8966(92)90004-p

[40] Hirst, R. C., McCullagh, K. J. & Davies, K. E. Utrophin upregulation in Duchenne muscular dystrophy. *Acta Myol ***24**, 209–216 (2005). 16629055

[41] AC’t Hoen, P. et al. Generation and characterization of transgenic mice with the full-length human DMD gene. *J. Biol. Chem. ***283**, 5899–5907 (2008). 18083704 10.1074/jbc.M709410200

[42] Valadares, M. et al. Human adipose tissue derived pericytes increase life span in Utrn tm1Ked Dmd mdx/J mice. *Stem Cell. Rev. Rep. ***10**, 830–840 (2014). 24943487 10.1007/s12015-014-9537-9

[43] Wang, Q. et al. A method of utrophin up-regulation through RNAi-mediated knockdown of the transcription factor EN1. *J. Int. Med. Res. ***39**, 161–171 (2011). 21672318 10.1177/147323001103900117

[44] Quattrocelli, M. et al. Long-term miR‐669a therapy alleviates chronic dilated cardiomyopathy in dystrophic mice. *J. Am. Heart Assoc. ***2**, e000284 (2013). 23963759 10.1161/JAHA.113.000284 PMC3828786

[45] Robinson, M. D., McCarthy, D. J. & Smyth, G. K. edgeR: a Bioconductor package for differential expression analysis of digital gene expression data. *Bioinformatics ***26**, 139–140 (2010). 19910308 10.1093/bioinformatics/btp616 PMC2796818

[46] Dai, X. & Zhao, P. X. psRNATarget: a plant small RNA target analysis server. *Nucleic Acids Res. ***39**, W155–W159 (2011). 21622958 10.1093/nar/gkr319 PMC3125753

[47] Chang, L. & Xia, J. *Transcription Factor Regulatory Networks* 185–204 (Springer, 2022).

[48] Chin, C. H. et al. cytoHubba: identifying hub objects and sub-networks from complex interactome. *BMC Syst. Biol. ***8**(Suppl 4), 896. 10.1186/1752-0509-8-s4-s11 (2014). 10.1186/1752-0509-8-S4-S11 PMC4290687 25521941

